# Reproductive biology, stem cells biotechnology and regenerative medicine: a 1-day national symposium held at Shahid Sadoughi University of Medical Sciences

**Published:** 2016-09

**Authors:** Fatemeh Akyash, Somayyeh Sadat Tahajjodi, Fatemeh Sadeghian-Nodoushan, Abbas Aflatoonian, Ali-Mohammad Abdoli, Habib Nikukar, Behrouz Aflatoonian

**Affiliations:** 1 *Stem Cell Biology Research Center, Yazd Reproductive Sciences Institute, Shahid Sadoughi University of Medical Sciences, Yazd, Iran.*; 2 *Research and Clinical Center for Infertility, Yazd Reproductive Sciences Institute, Shahid Sadoughi University of Medical Sciences, Yazd, Iran.*; 3 *Department of Advanced Medical Sciences and Technologies, School of Paramedicine, Shahid Sadoughi University of Medical Sciences, Yazd, Iran* *.*

**Keywords:** *Reproductive Biology*, *Stem Cell Biotechnology*, *Regenerative Medicine*, *Stem Cells*, *Germ Cells*

## Abstract

This paper summarizes the proceedings of a 1 day national symposium entitled “Reproductive biology, stem cells biotechnology and regenerative medicine” held at Shahid Sadoughi University of Medical Sciences, Yazd, Iran on 3^rd^ March 2016. Here, we collected the papers that presented and discussed at this meeting by specialists that currently researched about the overlaps of the fields of reproductive biology and stem cells and their applications in regenerative medicine.

The well-organized and on time reconstitution capability of the cells and tissues of the reproductive tract as an organ system confirm the existence of a relatively high ratio of stem cells. The main locations in female and male reproductive system which stem cells reside and can be either generated or isolated are the ovary, endometrium, decidua, and the testis. The cells derived from these tissues include oocytes, embryos, embryonic stem cells (ESCs), oogonial stem cells, endometrial mesenchymal stem cells (EnMSCs), trophoblast cells, and spermatogonial stem cells (SSCs) (1). Growing the number of the international reports on this area during last decade highlights the scientific importance of the field.

A recent 1-day national symposium entitled “Reproductive biology, stem cells biotechnology and regenerative medicine”, sponsored by the Stem Cell Biology Research Center, Yazd Reproductive Sciences Institute, Yazd Province Organization of Academic Center for Education, Culture and Research (ACECR), Council for Development of Stem Cell Sciences and Technologies (CSCT), Presidency of the Islamic Republic of Iran Vice-Presidency for Science and Technology, and hosted by the Shahid Sadoughi University of Medical Sciences, brought together specialists that researched about the overlaps of the fields of reproductive biology and stem cells and their applications in regenerative medicine on 3^rd^ March 2016. 

Here, we collected the papers that presented and discussed at this meeting. This symposium was divided into three sessions: The first session was about the generation and isolation of stem cells from reproductive system sources. The second session was about the differentiation of stem cells to reproductive cells. In the third session, specialists discussed about the ethics and tissue engineering and the future regenerative medicine applications in reproductive tract.

At the beginning of the scientific sessions, Shoae-Hassani on behalf of Hamidieh discussed about the stem cells and reproductive medicine and mentioned that “investigation in stem cells and regenerative medicine is greatening more and more, and its translation to the clinic is going fast by the recent massive volume of clinical trials. Over the past decade, scientists have advanced the field of reproductive tissue engineering to restore normal sexual function and preserve fertility in both female and male patients”. Moreover, they previously reported that many of reproductive derived stem cells could find clinical application in the future (2). 

Another paper by Baharvand described the derivation of ESCs and embryonic germ cells (EGCs) and their use in germ cell differentiation (3-5). 

In continues, Movahedin talked about the importance of SSCs in male reproduction and spermatogenesis. She demonstrated that only 0.03% of all germ cells include SSCs. SSCs transplantation can be used in cancer patients who had chemo/radiotherapy. Irreversible azoospermia is one of the side effects in these patients that can affect their quality of life seriously. Therefore, optimizing of culture’s conditions and enrichment of cryopreserved SSCs are main goals of male reproductive medicine (6). 

One of the populations of stem cells in female reproductive system resides in endometrium. Another paper by Salehnia described about isolation and characterization of EnMSCs, and their capability for self-renewal, colony-forming units (CFU), differentiation into mesoderm-derived lineages including adipogenic, myogenic, chondrogenic, osteogenic and expression of CD146, CD90, CD73, CD105, SOX2 markers and the lack of CD34 and CD14 marker expression (7). Similarly, other groups confirmed the isolation and characterization of stem cells from endometrium (8). 

Nematollahi-Mahani discussed about the role of umbilical cord cells in repair and improvement of reproductive system and their potential to differentiate into germ cells for using as an appropriate candidate in cell therapy strategies. Nematollahi-Mahani and co-workers in their paper indicated that human umbilical cord mesenchymal (hUCM) cells can be differentiated into the male germ cell *in vitro* (9).

Kalantar presented that human amniocytes appear to be multipotent in nature. These cells capable to *in vitro* differentiate into ectodermal and mesodermal cell types, which show their inherent plasticity. Also, other groups confirmed in their studies that amniocytes have stem cells characteristics (10). 

In the second session, Moghaddam-Matin mentioned the application of cytotrophoblast stem cells lines derived from human ESCs (hESCs) as a model for early human trophoblast invasion studies. In addition, trophoblast bodies closely mimicked early invasive stages of implantation when incubated with human endometrial stroma *in vitro* and these cell lines could be a significant new model for investigating of human placentation and may have applications in cell therapy (11). 

Shahverdi discussed about “genetic causes of infertility among men, most commonly due to the de novo deletion of one or more Azoospermia Factor (AZF) regions of the human Y chromosome. Pure sterile phenotypes in men with deletions vary from the complete absence of germ cells and sperm (termed Sertoli Cell Only syndrome; SCO) to production of germ cells that arrest in development (early maturation arrest; EMA) to very low sperm counts (oligospermia). Of new therapies, stem cells have opened new window for infertility treatments". In this regard, others reported that hESCs as pluripotent cells could differentiate into post meiotic male germ cells (12). 

Bahmanpour presented about mouse ESCs (mESCs) differentiation into oocyte- like cells with ovarian somatic cell co-culturing in combination by bone morphogenic protein 4 (BMP4) and retinoic acid (RA) supplementation (13). Bahmanpour mentioned *in vitro *ESCs derived gametes provides powerful model for understanding of epigenetic modifications during gametes development, causes of infertility and other molecular base of reproductive disorders. 

One way to obtain the functional oocyte (MII) is *in vitro* maturation (IVM) of immature (GV and MI) oocytes. In this regards, Khalili described about IVM of immature oocytes as one of the choices for fertility preservation. He suggested that future studies focused on improvement of the oocyte maturation procedure, like co-culture system for infertile patients with PCOs or young cases with life circumstances (cancer). These oocytes represent pool of germ cells that can be matured and used in assisted reproductive program (14). 

In the last session, Haghirosadat discussed about the general ethical issues in the field of reproductive biology and stem cells according to the Islamic rules and international laws (15). 

Arjmand discussed about stem cells translational medicine and mentioned that stem cell therapy has introduced promising hopes for the treatment of various diseases. But, during this translation, quality level should be performed based on good manufacturing practice (GMP) standards and rules (16). 

Niknejad described that the amniotic membrane (AM), with unique characteristics and natural biomaterial components in extra cellular matrix (ECM) of basement membrane including collagen, fibronectin, laminin and other proteoglycans could be used as a natural scaffold in tissue engineering (17). 

In continuous, Kajbafzadeh discussed about tissue engineering and regenerative medicine of reproductive tissues and organs. Furthermore, he mentioned the natural history of reproductive system decellularization, preserved ECM and in situ scaffold recellularization/cell seeding, differentiation of umbilical cord stem cells into germ cells, vagina and urethral reconstruction by ECM (18). 

In the field of female reproductive tissue engineering, Nikukar mentioned malfunctioned of main organs are the causes of transient or constant infertility and artificial or organic scaffolds as basement membranes can carry and affect the selected pluripotent stem cells. It is not so far unpredictable that you could creative an organ or a tissue by prescription of your physician. But the gap between laboratory tests and clinical applications is deep enough to use the regeneration as a treatment technique for the patients (19). 

As an example of the future possible application of tissue engineering in male reproductive regenerative medicine, Borzouie presented the data of her Ph.D. thesis which was about the development of an artificial human testis using a novel 3D culture device applying a home-made scaffold. However, the research was in preliminary stages but it could lend itself into the future cell therapy applications in the field of male infertility (20). 

Finally, Aflatoonian discussed about the derivation of Yazd hESCs (Aflatoonian et al, unpublished data) and mentioned about the future applications of hESCs in regenerative medicine. hESCs with pluripotency capacity could differentiate into every cell types derived from three embryonic germ layers such as, neurons, cardiomyocytes, hepatocytes, hair cells, retinal pigmented epithelial (RPE) cells, and even post meiotic germ cells. Moreover, in vivo animal model studies are performing to modify the capacity of the hESCs-derived cells in regenerative medicine (21). Efforts have begun to generate clinical grade hESCs for their future therapeutic applications (22).
